# Unidirectional airflow, air sacs or the horizontal septum: what does it take to make a bird lung?

**DOI:** 10.1098/rstb.2023.0418

**Published:** 2025-02-27

**Authors:** Emma R. Schachner, Andrew J. Moore

**Affiliations:** ^1^ Department of Physiological Sciences, College of Veterinary Medicine, University of Florida, Gainesville, FL 32603, USA; ^2^ Department of Anatomical Sciences, Renaissance School of Medicine, Stony Brook University, Stony Brook, NY 11794, USA

**Keywords:** respiratory, Aves, Sauropsida, pulmonary, evolution, pneumaticity

## Abstract

In this review, we evaluate the differences between the pulmonary anatomy of birds and other sauropsids, specifically those traits that make the avian respiratory system distinct: a fully decoupled and immobilized, isovolumetric gas-exchanging lung separated from compliant ventilatory air sacs by a horizontal septum. Imaging data, three-dimensional digital anatomical models and dissection images from a red-tailed hawk (*Buteo jamaicensis*), common ostrich (*Struthio camelus*), barred owl (*Strix varia*), African grey parrot (*Psittacus erithacus*) and zebra finch (*Taeniopygia castanotis*) are used to demonstrate the anatomical variation seen in the pulmonary air sacs, diverticula and the horizontal septum. We address the current state of knowledge regarding the avian respiratory system and the myriad areas that require further study, including the comparative and quantitative ecomorphology of the bronchial tree and air sacs, the non-ventilatory functions of the sacs and diverticula, the fluid dynamics and anatomical mechanisms underlying unidirectional airflow, post-cranial skeletal pneumaticity, and how all of these factors impact reconstructions of respiratory tissues in extinct archosaurs, particularly ornithodirans (i.e. pterosaurs + non-avian dinosaurs). Specifically, we argue that without evidence for the horizontal septum, a fully avian lung should not be reconstructed in non-avian ornithodirans, despite the presence of post-cranial skeletal pneumaticity.

This article is part of the theme issue ‘The biology of the avian respiratory system’.

## 1. Introduction

The origin and evolution of the functionally decoupled avian respiratory system is one of the most enduring questions in vertebrate biology, with the first known description of avian pulmonary anatomy published in 1573 [1]. Despite almost 450 years of research, this organ system remains as fascinating to biologists as it was during the sixteenth century. The anatomical and functional divergence between the bronchoalveolar mammalian lung and the heterogeneously partitioned sauropsid lung remains poorly understood, as do the specific innovations that differentiate birds from their extinct and extant non-avian sauropsid relatives. Advances in three-dimensional imaging allow for unprecedented evaluation of pulmonary evolution and diversity and are critical for characterizing the various evolutionary modifications along the ‘reptile’ lineage and visualizing delicate structures in the avian lung that remain difficult to study *in situ*.

In this review, we summarize the current state of knowledge on the avian respiratory system and the structural differences that distinguish avian and non-avian sauropsids, with a focus on air sac diverticula, the horizontal septum and unidirectional flow. In addition, we use both classical dissection techniques and three-dimensional anatomical segmentation of micro-computed tomography (µCT) scans to showcase areas of active study and to provide novel data demonstrating aspects of the avian respiratory system that remain largely unexplored. We close by highlighting specific topics in need of further inquiry and discussing how new insights and outstanding uncertainty impact reconstructions of the respiratory system in extinct archosaurs.

## 2. Material and methods

Centuries of anatomical work on birds and other sauropsids have produced a diverse nomenclature for structures in their lower respiratory systems, with partially overlapping sets of terminology implying homologies that have not yet been demonstrated (e.g. by phylogenetic or developmental studies). Here we follow Duncker [2] for most of the pulmonary structures of birds and Schachner *et al*. [3–5] for the non-avian taxa. We also follow the *Nomina Anatomica Avium* [6] in applying cranial/caudal (as opposed to anterior/posterior) as post-cranial directional terms.

One red-tailed hawk (*Buteo jamaicensis*) and two barred owls (*Strix varia*) were collected as salvage specimens on US Fish and Wildlife Scientific Collecting permit to ERS no. MBPER8930896. One owl was donated by the Auburn Raptor Center (*S. varia*, unnamed adult, sex unknown, ERS2019-027), one hawk was obtained as a donation from a local veterinary clinic (*B. jamaicensis*, Alexander, sub-adult female, ERS2021-001) and the second owl (*S. varia*, Thucydides, adult sex unknown, ERS2024-007) was obtained from Zoological Medicine at the University of Florida College of Veterinary Medicine. Computed tomography (CT) imaging data from one specimen of a Cuvier’s dwarf caiman (*Paleosuchus palpebrosus*) were used from Schachner *et al*. [3] (specimen ERS2020-003, Quintus Sertorius). This individual was imaged while sedated for clinical purposes unrelated to the study (adult male, approximately 40−45 years old), and weighed 12 kg at the time of the scan. He was imaged on a GE Medical Systems LightSpeed QX/I scanner at the San Antonio Zoo. CT imaging data from one specimen of a common ostrich (*Struthio camelus*) were used from Schachner *et al*. [7]: specimen CS 11. This specimen was deceased (sex unknown), weighed 71.3 kg and imaged at the Royal Veterinary College London.

One owl (ERS2019-027) was dissected using standard techniques to demonstrate the horizontal septum. To access this septum, the interclavicular, cranial thoracic and caudal thoracic air sacs were all ruptured; however, the abdominal air sacs retained some air at the equivalent of an end tidal volume (see fig. 5 in Lawson *et al*. [8]). The *B. jamaicensis* specimen and one *S. varia* were imaged at the Research Service Center of the Herbert Wertheim College of Engineering at the University of Florida (RRID:SCR_025135). The *S. varia* specimen (Thucydides, ERS2024-007) was visualized in the multi-planar reconstruction (MPR) viewer in OsiriX MD (https://www.osirix-viewer.com/) and the axes were aligned parallel and perpendicular with the thoracic vertebrae in sagittal and coronal view following the methods of Schachner *et al*. [9], to visualize the horizontal septum. The lower respiratory system of a specimen of a *B. jamaicensis* (Alexander) and one specimen of *P. palpebrosus* were segmented following the methods of Schachner *et al*. [4] with the scientific visualization program Avizo 2023 (Thermo Fisher Scientific).

## 3. The structure and functional anatomy of the avian lung

### (a) The gas-exchanging lung and bronchial tree

**Figure 1.**
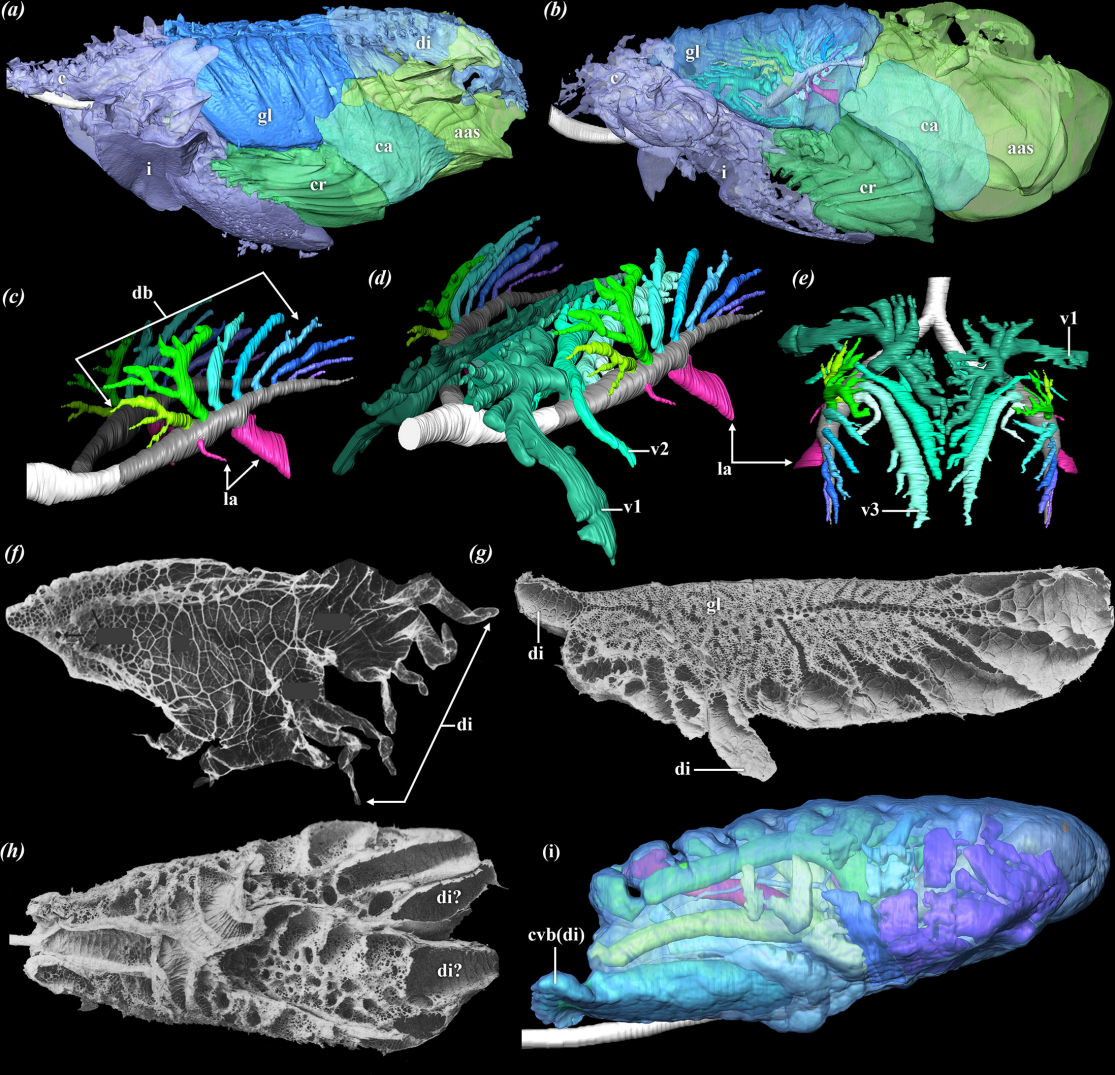
Lungs morphology across Sauropsida**.** Left lateral view of the gas-exchanging lung and air sacs of (*a*) a red-tailed hawk (*B. jamaicensis*) and (*b*) African grey parrot (*Psittacus erithacus*; modified from Lawson *et al*. [16]) segmented from µCT data. Solid representation of the bronchial tree (negative space) of the common ostrich (*S. camelus*) segmented from µCT data, modified from Schachner *et al.* [7] in left craniolateral (*c, d*) and dorsal (*e*) views. (*f*) Medial view of the inflated lungs of the common chameleon (*Chamaeleo chamaeleon*), modified from Perry & Duncker [17]. (*g*) Medial view of the lungs of the lace monitor (*Varanus varius*) and (*h*) ventral (opened) view of the lungs of the Bengal monitor (*V. bengalensus*), modified from Milani [18]. (*i*) Left lateral view of a segmented model of the lungs and bronchial tree of Cuvier’s dwarf caiman (*Paleosuchus palpebrosus*). Images not to scale. Abbreviations: aas, abdominal air sac; c, cervical air sac; ca, caudal thoracic air sac; cr, cranial thoracic air sac; cvb(di), cervical ventral bronchus—diverticulum; db, dorsobronchi; di, diverticulum; gl, gas-exchanging lung; i, interclavicular air sac; la, laterobronchus (lateroventral secondary bronchi); v, ventrobronchus.

The avian respiratory system is composed of a fully heterogeneous system in which the gas-exchanger is completely decoupled from the ventilator [10–14]. While the entire system is technically all ‘lung’ or pulmonary tissues, the term ‘lung’ is often used to refer solely to the gas-exchanging portion (e.g. [15]). For clarity, we refer to the latter as the ‘gas-exchanging lung’ or ‘exchange parenchyma’ to distinguish it from the ventilatory air sac components of the pulmonary system (figure 1*a*,*b*).

**Figure 2.**
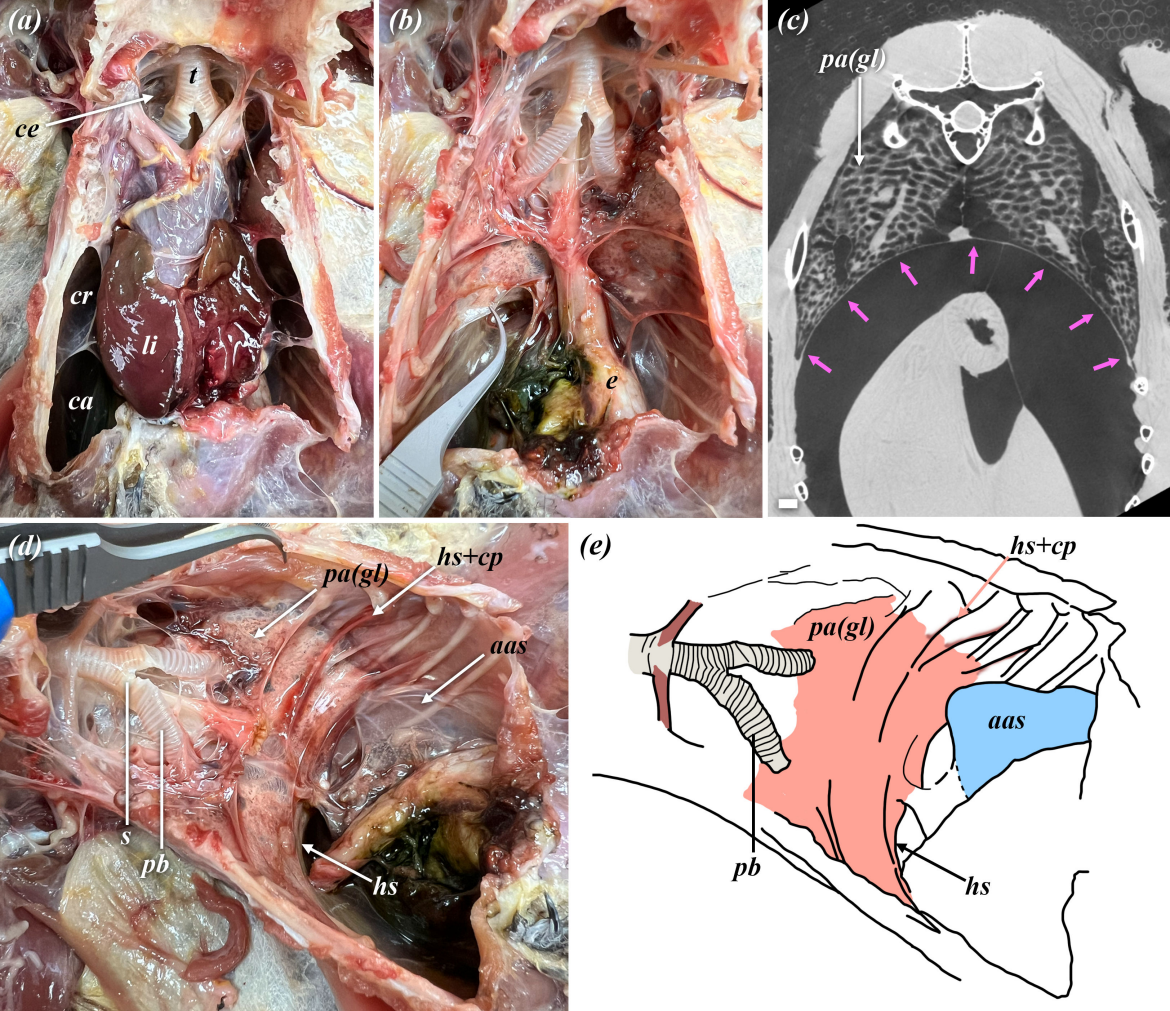
Anatomy of the horizontal septum in the barred owl (*S. varia*). (*a*) Coelomic cavity of *S. varia* in ventral view with the pericardium, liver, trachea and ruptured interclavicular, cranial thoracic and caudal thoracic air sacs exposed. (*b*) Viscera have been removed exposing the ventral surface of the gas-exchanging lung and horizontal septum (gripped by tweezers). (*c*) µCT image of an adult *S. varia* gas-exchanging lung demonstrating the horizontal septum (indicated by the pink arrows); scale bar = 1 cm. (*d*) Gas-exchanging lung shown in oblique ventrolateral view, demonstrating the horizontal septum, the lung hilus, its attachment to the medial aspect of the dorsal ribs, along with the left abdominal air sac extending through the opening in the septum into the coelomic cavity with the abdominal viscera. (*e*) Diagrammatic illustration of (*d*). Abbreviations: aas, abdominal air sac; ca, caudal thoracic air sac; cp, costopulmonales muscles; ce, cervical air sac; cr, cranial thoracic air sac; e, esophagus; hs, horizontal septum; li, liver; pa(gl), parabronchi (gas-exchanging lung); pb, primary bronchus; t, trachea.

Unlike other sauropsids, the gas-exchanging lung in birds is completely immobilized and gas exchange occurs via a cross-current system [19]. The exchange parenchyma is bounded dorsally and incised by the vertebral column and ribs, and is ventrally constrained by the horizontal septum [2,20,21] (figure 2); for diagrammatic images and illustrations, see fig. 13*b* of Schachner *et al*. [22] and fig. 2.14 of Duncker [23]. This septum contains interspecifically variable paired muscles, the costopulmonales, which run from the medial aspect of the sternal component of the junction between the sternal and dorsal ribs to attach to the aponeurosis of the septum and ventral spinous processes (hypapophyses) [20,24]. Electromyogram data indicate that these muscles contract during expiration, and it has been suggested that they play a critical role in maintaining the integrity of the air sac ostia and the immobility of the gas-exchanging lung [20,24,25]. Immobility of the gas-exchanging lung allows for the fusion of the parietal and visceral pleurae and the effective obliteration of the pleural cavity during the later stages of development [21,26]. The avian gas-exchanging lung is often described as ‘rigid’ but this is not strictly correct. The exchange parenchyma is not cartilaginous or ossified, and undergoes minute changes in volume during the respiratory cycle (e.g. 1.4% in Pekin ducks (*Anas platyrhynchos*) [25]). These volumetric changes are functionally insignificant relative to other sauropsids, for which the exchange parenchyma may experience a resting-to-maximum lung volume size fluctuation up to 80% in some species (e.g. 80% in the common chameleon (*Chamaeleo chamaeleon*) and 76% in the savanna monitor lizard (*Varanus exanthematicus*) [17]). For this reason, the gas-exchanging lung in birds can be described as essentially isovolumetric and had likely evolved in Mesozoic birds by at least the Late Cretaceous [27].

The isovolumetric immobility of the avian lung has been proposed as an evolutionary prerequisite for the dramatic thinning of the blood−gas barrier (BGB) [28], and allows for an increase in respiratory surface area [29], both of which are among the structural necessities that provide birds with the ability to meet the oxygen demand required for active powered flight [30–36]. Maina & West [34] found that the harmonic mean thickness of the BGB (a measure of the oxygen diffusion capacity of the gas-exchanging lung) in birds was smaller than in mammals by a factor of 2.5, even in similarly sized animals. Birds also have approximately a 15% larger BGB surface area relative to mammals of a similar body mass [37], which is structurally sustainable due to the strengthening presence of type IV collagen in the extracellular matrix [38]. Experimental disruption of the horizontal septum results in deformation of the gas-exchanging lung and death of the bird [20], underscoring the critical role of this tissue in the maintenance of gas-exchanging lung immobility and functionality, perhaps by safeguarding against BGB collapse [31]. Thoracic ribs that bound and incise the gas-exchanging lung are also essential for parenchymal immobility. This skeletal infrastructure first appeared among the dinosauromorph ancestors of birds and has been implicated as a crucial prerequisite for the evolutionary thinning of the BGB [28,39–41], which may have facilitated the success of bird-line archosaurs in the low atmospheric oxygen environments of the Late Triassic [39,40].

The bronchial tree of birds begins with a trachea of varying length, occasionally with a long and looping segment that coils deep into a sternal excavation, as in the red-crowned crane (*Grus japonensis*), whooping crane (*Grus americana*), tundra swan (*Cygnus columbianus*) and trumpeter swan (*Cygnus buccinator*; e.g. [42–44]) or wraps as far caudally as the cloaca in curl-crested manucodes (*Manucodia comrii*), before returning up to the thoracic inlet [45]. This tracheal looping morphology has also been found to be present in crocodilians [46,47]. The trachea bifurcates at the carina, which is where the avian organ of sound production sits as a tracheal syrinx, a tracheobronchial syrinx (as in most taxa) or bronchial syrinxes (as in, e.g. king penguins (*Aptenodytes patagonicus*)) positioned at the origin of each primary bronchus (e.g. [48–50]).

The intrapulmonary primary bronchus first gives off a series (typically 4−5) of medially projecting ventrobronchi (also called medioventral secondary bronchi). These are followed by a short diastema, or gap, after which arise 5−10 dorsobronchi (also called mediodorsal secondary bronchi) [2,15,16,33,51] (figure 1*c*–e). The dorsobronchi arise sequentially, branching into a highly organized and ordered parabronchial network—the paleopulmonic parabronchi or paleopulmo—which connects directly with the ventrobronchi to form a continuous loop [2,15,16,33,51]. The paleopulmo is where the cross-current gas-exchange occurs [19]. In most taxa described to date, the parabronchi anastomose extensively in their arching trajectory between the dorsobronchi and ventrobronchi [2]. Interestingly, in *Taeniopygia castanotis*, individual parabronchi connect specific dorsobronchi to individual ventrobronchi and mostly lack substantial inter-parabronchial anastomoses, but the impact on respiratory function remains obscure [52].

Lateral and ventral to the dorsobronchi is an aggregation of disorganized parabronchi called the neopulmo. The neopulmo is not present in all birds, but in many species can occupy up to 20% of the mass of the gas-exchanging lung [2]. In taxa with a more substantial and developed neopulmo, like songbirds, the primary bronchus and secondary bronchi are positioned more medially [51], with the neopulmo occupying most of the lateral aspect of the gas-exchanging lung surface (see, e.g. the bronchial tree and neopulmo of the zebra finch (*T. castanotis*; [52, fig. 2b])). The functional significance of the neopulmo, relative to the paleopulmonic parabronchi, remains to be determined.

The laterobronchi are the third series of highly variable secondary airways that branch from the primary bronchus, projecting ventrally (sometimes identified as the lateroventral secondary bronchi) and laterally (also identified as the laterodorsal secondary bronchi) into the neopulmo and usually comprising one large laterobronchus that directly connects to the caudal thoracic air sac [7,16,51] (figure 1*c*–e). While the generalized map of the bronchial tree has been well established using dissection, latex and other injection-based methods (e.g. [2,53]), as well as more recently by imaging-based methods (e.g. [4,7,16,52,54]), clade-level ecological and evolutionary analyses have not yet been undertaken in the same way that they have for, e.g. avian skeletal elements (e.g. [55,56]).

### (b) The ventilatory air sacs

Extending from the bronchial tree beyond the cranial, ventrolateral and caudal borders of the gas-exchanging lung are a series of generally nine air sacs that serve primarily as ventilatory bellows [2,13,15,37,57–60]. These include the unpaired interclavicular sac and paired cervical, cranial thoracic, caudal thoracic and abdominal air sacs (figure 1*a*,*b*). Following a general pattern, the cervical sacs branch off of the first ventrobronchus and extend beyond the cranial margin of the gas-exchanging lung towards the cervical vertebrae, while the cranial thoracic sacs expand from the third ventrobronchus and pass ventrally through the horizontal septum towards the sternum. The caudal thoracic sacs branch off of the large, ventrally projecting laterobronchus, opposing the dorsobronchi, and, like the cranial thoracic sacs, pass ventrally through the horizontal septum and expand caudally.

All of the sacs with the exception of the abdominal sacs lie between the horizontal and oblique septa in the subpulmonary space and are separated from the abdominal viscera [2,61]. The abdominal sacs emerge as an expansion of the intrapulmonary primary bronchus (but see Martinez *et al*. [52] for a variant of the origin of the abdominal sac in zebra finches) [2,7,16,32,33]. With certain variations, the air sacs are developmental dilations of the terminal parts of the airways. Specifically, the intrapulmonary primary bronchus and secondary airways serve as direct ostial connections to the sacs, with indirect connections via the parabronchi [21,59,62,63]. The opening into the abdominal air sac passes through an ostium in the caudal margin of the horizontal septum and the sac extends into the coelomic cavity to share space with the abdominal organs [21]. As described above, the remaining sacs are contained in the subpulmonary space—that is, the space bounded dorsally by the horizontal septum and caudally by the oblique septum [2]. The latter structure originates from the dorsal mesentery, continues as an extension of the horizontal septum caudally, and inserts laterally along the dorsal border of the sternum [2,58]. The oblique septum has been proposed as a potential homologous structure to the postpulmonary septum in squamates [61]. As previously described for the bronchial tree, specific avian air sacs have also been proposed to be both functionally and structurally homologous to regions of the lungs in crocodilians [7,22,64] based upon similarities in bronchus development [62,63,65] and airflow patterns [46,66,67].

**Figure 3.**
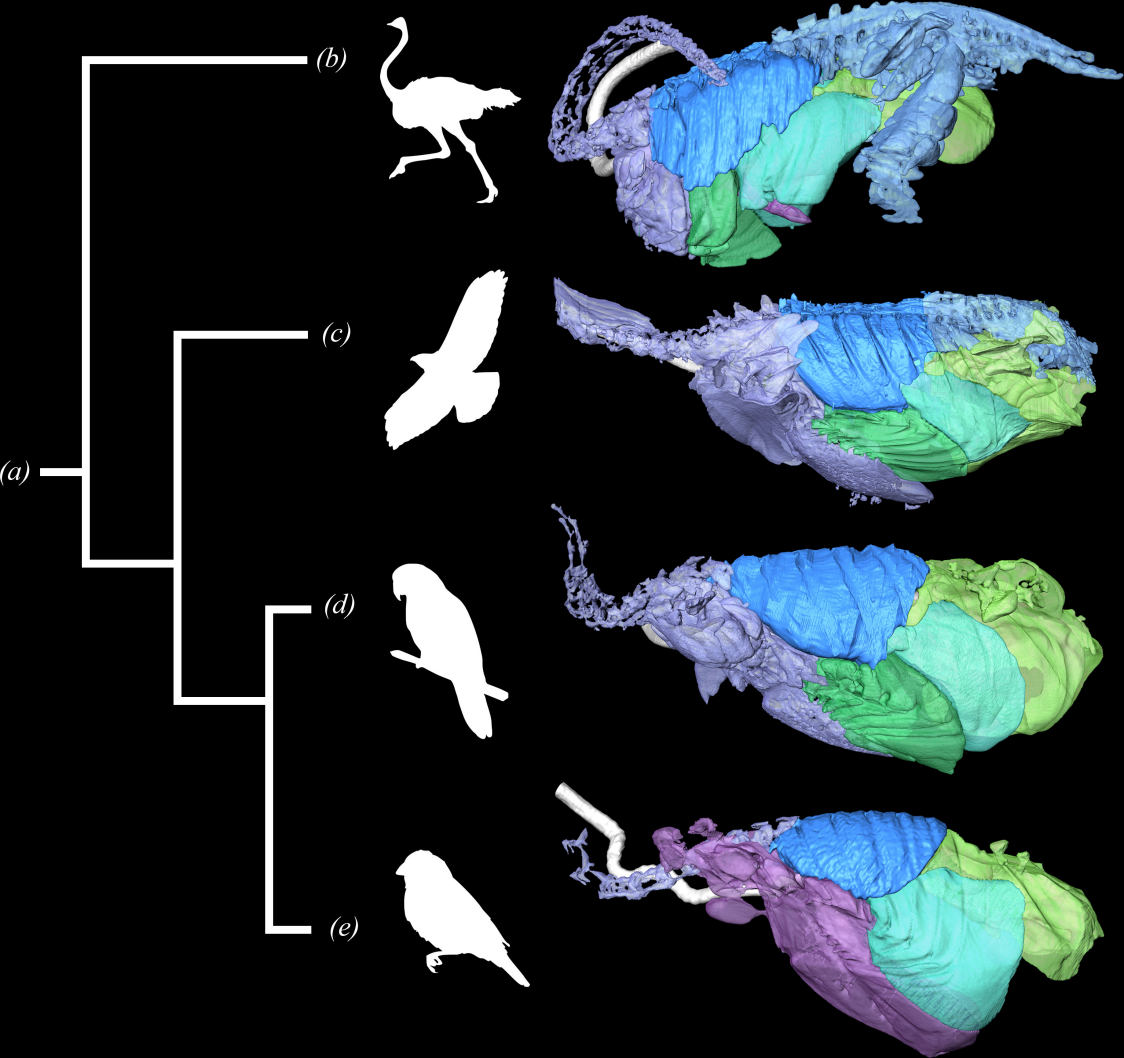
Phylogeny for Aves demonstrating the structural diversity in the air sacs and pulmonary diverticula. (*a*) Aves. (*b*) *Struthio camelus* segmented lung model modified from Schachner *et al.* [7]. (*c*) *Buteo jamaicensis* segmented lung model. (*d*) *Psittacus erithacus* segmented lung model modified from Lawson *et al.* [16]. (*e*) *Taeniopygia castanotis* modified from Martinez *et al.* [52]. All lung models in left lateral view and images not to scale. Silhouettes from PhyloPic.

Intraspecific and interspecific air sac morphology is highly variable (figure 3) [2]. Lawson *et al*. [8] evaluated the air sacs of naturally deceased as well as live sedated African grey parrots (*P. erithacus*) using three-dimensional anatomical modelling and found that while all of the specimens shared an overall similarity in their air sac pattern, there was considerable intraspecific variation in the extent and size of some of the individual sacs. Noteworthy variation included how far caudally the interclavicular sac extended along the internal surface of the sternum and the bilateral asymmetry of some of the sacs [8]. Some passerines, hummingbirds and kiwi possess relatively small abdominal air sacs [2,52,53,68], while galliforms and anseriforms have large ones [2,69]. Many taxa have been found to fuse individual sacs as adults [58]. For example, fusion of the interclavicular with the cranial thoracic sac occurs regularly in passerines [2,52] and *B. jamaicensis.* Scheid *et al*. [70] found differential ventilation of individual sacs under experimental analysis in adult ducks (*A. platyrhynchos*), suggesting that interspecific variation in sac fusion should impact airflow patterns and mixing of gases. However, these potential fusion-induced alterations in air flow have yet to be experimentally characterized.

Non-ventilatory functions of the air sacs have been less well studied. Considering that crocodilians shift their lungs within their coelomic cavity [71] to actively control their centre of mass while diving [72], it is highly possible that birds are using their ventilatory air sacs (as well as their non-ventilatory diverticula; see below) for myriad secondary roles as well, perhaps even locomotory behaviours. Birds can voluntarily collapse specific individual sacs, like the abdominal sac [2,8,60], or diverticula extending from a sac, like the subpectoral diverticulum (SPD), and may do so asymmetrically or bilaterally [9]. This capacity for individualized control of at least some portions of the air sac system suggests functional differentiation. The interclavicular sac has been demonstrated to have a functional relationship with vocalization in many taxa. In mallards (*A. platyrhynchos*), the interclavicular sac has been experimentally recorded as having increased internal pressures during vocalization [73]. Starlings (*Sturnus vulgaris*) lose the ability to vocalize if the interclavicular sac is ruptured and show dramatic increases in air sac pressures during distress calls [74] suggesting an active role for the air sacs in modulating vocalization.

The air sacs have occasionally been suggested to play a role in thermoregulation. The abdominal air sacs in birds were proposed to serve as a mechanism for lowering the temperature of the testes to prevent heat damage during spermatogenesis [75]; however, this was later demonstrated to be false through experimental work in the domestic fowl (*Gallus gallus*) [76]. Notably, the roosters used in the latter study had their abdominal air sacs surgically removed (*n* = 34, 17 non-surgical controls), yet survived the procedure and were able to recover and produce sperm for the experiment [76]. While not the aim of the study, this suggests that, despite often being the largest air sacs, the abdominal sacs may be physiologically less critical for ventilation than other regions of the lung, at least in *G. gallus*, and perhaps have additional, undiscovered functions. A second and equally contentious thermoregulatory hypothesis for the air sacs is that they modulate whole-body temperature through evaporative cooling, especially during panting (e.g. [24,77–79]). There have been a few experimental studies suggesting that this may be a possibility [80,81]. However, work in *G. gallus* by Menuam & Richards [82] measured the temperatures of several individual sacs as well as surfaces of the upper respiratory tract during hyperthermia and found that the trachea and the nasal and buccal regions act as important sites of evaporative cooling whereas the examined air sacs were not appreciably involved. Without further examination, the involvement of the air sacs in evaporative cooling remains equivocal.

Air sac innervation and vascularization is an area of study that has only sporadically been addressed. The air sacs are generally considered to have a negligible vascular supply, but an interspecific phylogenetic survey has never been completed and endoscopic images of the cranial thoracic air sac in a live hybrid falcon show a clear vascular network in the air sac walls [83]. There are some data demonstrating that a ring of smooth muscle surrounds the ostia that connect the abdominal and caudal thoracic sacs to the secondary bronchi [84]. These sphincters are proposed be under autonomic control (adrenergic) [84,85], but the functional significance of the sphincters themselves has not been conclusively established. Innervation of the air sacs by the vagus nerve (CNX) has been described for the zebra finch (*T. castanotis*) [86] and unidentified nerves supplying the horizontal septum (sometimes termed the saccopleural membrane) are present in the domestic fowl (*G. gallus*) [87]. Watanabe *et al*. [88] described parasympathetic neurons as ‘sparsely present’ in the air sacs of embryonic chick air sacs, with the ganglia retained within the gas-exchanging lung. An adrenergic plexus has been described as being present in association with a vascular plexus in the air sacs of chickens (*G. gallus*) and the mute swan (*Cygnus olor*), along with observable interspecific differences [89]. We identified a potentially large nerve plexus extending around the proximoventral surface of the abdominal sac in an adult barred owl (*S. varia*) (figure 2*d*), just distal to where it emerges from the gas-exchanging lung, and while likely also vagal branches, the type of fibres present in these nerves were not determined. Bennett & Malmfors [85] observed that while infrequent, non-terminal axon bundles have been observed in the walls of the ventilatory air sacs in the domestic fowl, particularly the abdominal sac, but they did not elaborate further on the topic. It has been proposed that the sacs contain both chemoreceptors and mechanoreceptors [86]; however, this has not been resolved and innervation of avian air sacs remains an area for future evaluation.

### (c) Non-ventilatory pulmonary diverticula

There are numerous non-ventilatory diverticula that emerge from the gas-exchanging lung and ventilatory air sacs of birds that have been described via anatomical dissection, injection-based casting and anatomical modelling [2,7,9,58,60]. These diverticula often directly pneumatize adjacent skeletal tissues (e.g. [60,90–92] and references therein), or traverse the body cavity and body wall, extending around organs and joints and between muscles. In some taxa, like pelicans and the turkey vulture, these diverticula occupy substantial subcutaneous space, and may extend to the distal aspect of the manus [2,93] or to the knee in the common ostrich [7,94]. These diverticula have been proposed to have numerous untested functions, including pneumatic bracing of joints [95], protection while plunging into the water [96,97], altering the specific gravity of diving foragers [96,98], functioning to reduce friction between organs or muscles, or playing a role in enhancing ventilation (e.g. [60,98–101]). Hamlet & Fisher [102] made the observation that the renal diverticula may play a role as a cushion around the kidneys and reproductive organs. These diverticula are common in many avian taxa, occupying the space between the kidneys and the synsacrum, and might therefore be functionally analogous to the perirenal fat pad in mammals. The supramedullary ( = paramedullary) diverticula have been suggested to play a similar cushioning role for the spinal cord [103].

There are very few examples of specific saccular or parabronchial diverticula (*sensu* Moore & Schachner [91]) being evaluated independently for function. One exception is the SPD, a large extension of the interclavicular sac that leaves the coelomic cavity and dives between the pectoralis and supracoracoideus muscles [58,98]. Schachner *et al*. [9] found that the SPD evolved independently in soaring birds at least seven times and was not present in taxa that used other flight modalities. They also found that the SPD was not integral for ventilation, as live sedated red-tailed hawks (*B. jamaicensis*) were able to bilaterally and unilaterally collapse the diverticulum. Additionally, through multibody dynamics analysis in *B. jamaicensis* and comparative muscle fascicle analysis, they found that the pectoralis has shorter muscle fascicles around the position of the SPD in hawks relative to non-soaring taxa and that an inflated SPD enhanced the torque generating ability of the pectoralis muscle. Collectively, these features—i.e. relatively short pectoralis muscle fascicles and the SPD—serve to specialize the flight apparatus for the isometric contraction required when the wing is held horizontally during soaring [9].

While the air sacs and their extraosseous diverticula are widely described as thin and poorly vascularized membranes (e.g. [60,90,94,100] and references therein), Müller [60] noted that pneumatizing diverticula are richly supplied by a capillary network derived from the systemic arterial circuit and asserted that these could represent sites of extrapulmonary gas exchange (albeit poor ones; see also [104,105]). Strasser [100] likewise remarked on the presence of vascular networks along the endosteal walls of pneumatic bones but asserted that these were well developed only in incompletely pneumatized bones and degenerate in fully pneumatic elements. The possibility that intraosseous vascular networks persist in pneumatized bones and potentially operate as sites of extrapulmonary gas exchange warrants further study.

## 4. Not-so-novel?: features of the avian respiratory system shared with other vertebrates

### (a) Air sacs and diverticula in non-avian sauropsids

The term ‘air sacs’ is usually reserved for avian sauropsids (birds), but there are many squamates with well-developed ventral or caudal regions of the lung that have historically been identified as air sacs, and it is difficult to find a distinct anatomical difference between these structures and the ventilatory air sacs of birds. Indeed, their similarity prompted Brattstrom [106] to make generalized comparisons between the ventilatory mechanics of snakes and birds, and others to speculate on the evolutionary origins of the avian air sacs within their squamate and crocodylian relatives [68,77,107]. Here, we provisionally identify any low parenchyma, sac-like extension of the lung as an air sac or diverticulum, pending comparative work establishing developmental homology.

With the exception of pythons, boas and a few other taxa, which retain a smaller vestigial left lung (ranging from 9 to 83% the size of the right lung in Booidea [108]), most snakes have one long right lung and restrict the gas-exchanging parenchyma to the cranial portion. The caudal region is instead developed as a sac-like structure with reduced vasculature and parenchymal density [108–110]. This caudal air sac has been proposed to function as (i) an accessory air reservoir for when large prey items are swallowed [111]; (ii) a flotation device (= swim bladder) [106,112–114]; (iii) a thermoregulatory structure for evaporative cooling [106]; (iv) a balloon to maintain the structural integrity of the body [106,111,115]; (v) a mechanism for enlarging the body during displays (not to be confused with the non-ventilatory hood-flaring of cobras) [108,114,116]; and (vi) a means of assisting in locomotion [115]. Very few of these hypotheses have been experimentally tested in a rigorous manner but are based on observations, such as the defensive expansive display of some arboreal colubrids [108,114]. Using XROMM coupled with electromyography, Capano *et al*. [117] demonstrated that boa constrictors (*Boa constrictor*) can modulate how they ventilate different regions of their lung—specifically that they were able to independently ventilate the vascularized and saccular regions while constricting.

Some species of ophidians who have secondarily lost their left lung, e.g. the king cobra (*Ophiophagus hannah*) and red-tailed green ratsnake (*Gonyosoma oxycephalum*), possess multiple, variable tracheal diverticula with no gas exchange tissue. These have been proposed to enhance hissing and growling [118–120], but like the saccular caudal region in snakes, the function of these diverticula has not been extensively studied or tested. Vipers, sea snakes and other species have a tracheal lung with respiratory parenchyma for enhancing gas-exchange [119,121]. Distinct from the tracheal diverticula of some elapids and colubrids, a tracheal lung is an expanded tracheal membrane with vascularized respiratory tissue [108]. This structure is present in many snake groups and is particularly well developed in, e.g. Viperidae and Hydrophiidae [108]. Like the non-respiratory diverticula in the trachea, the tracheal lung has been proposed to impact sound production in these taxa [120] but has not been well studied from a functional perspective (for a detailed discussion, see [108]).

Chameleons are another notable example of squamates in which the caudal region of the lung has developed into an extensive saccular region with reduced vascularity [17,122]. The respiratory parenchyma is aggregated in the cranial region of the lung and elaborate diverticula variably project caudally in a diverse array that wrap around the coelomic organs [17,18,122–125] (figure 1*f*). While the function of these diverticula remains unknown, they may be homologous with the saccular regions of other sauropsid lungs, including the caudal thoracic and abdominal air sacs of birds, a hypothesis proposed by Huxley [68] in his description of the air sacs of the brown kiwi (*Apteryx*).

Some species of varanids demonstrate distinct diverticula that project beyond the margin of the gas-exchanging lung [18,126]. Becker [126] noted that this is the case in quite a few taxa, including the lace monitor (*Varanus varius*) (figure 1*g*), mangrove monitor (*V. indicus*), Bengal monitor (*V. bengalensis*) (figure 1*h*), desert monitor (*V. griseus*) and crocodile monitor (*V. salvadorii*), among others. He observed that this morphology seems to correlate with large size, speculating that the diverticula may play a role in ventilation. However, these ‘extrapulmonary’ diverticula are not present in all large varanids, and their function and evolutionary distribution require further investigation.

Like squamates, crocodilians have regions of their lungs with reduced vascularization that, while distinct from the diverticular extensions described above, have been proposed to be homologous to avian air sacs [7,22,46,64,68]. Specifically, the caudoventral saccular region of the crocodilian lung has been proposed to be homologous to the abdominal sacs of birds, and the cranial margin of the cervical ventral bronchus (figure 1*i*) has been proposed to be homologous to the cervical air sac (see fig. 23 of Schachner *et al*. [7]). These modified pulmonary structures in non-avian sauropsid lungs indicate that the capacity for functional specialization of a heterogenous lung through the development of air sacs and non-ventilatory diverticula is not unique to birds. Instead, sauropsids appear to share a common developmental plasticity in the ability of their respiratory tissues to shift the parenchyma as well as generate new sites of sac-like enlargement.

### (b) Unidirectional airflow patterns

Hazelhoff [127] conceptualized the first models for the lungs of birds in which air flows in the same direction (i.e. caudal to cranial) during both phases of the respiratory cycle via aerodynamic valves. Later, experimental work using implanted flow probes and mass spectrometer gas analysis on live anesthetized and/or awake ducks and geese confirmed and refined these hypotheses, demonstrating that inspired air moved through the dorsobronchi and ventrobronchi in the same direction [10–13,128–131]. Early studies proposed that mechanical flaps or valves were involved in maintaining these flow patterns (e.g. [132]), but no evidence supporting this hypothesis has been found and all subsequent data indicate that airflow patterns are maintained by the angulation, shape and arrangement of the bronchial tree—that is, through aerodynamic valving [133–136].

What has emerged through both the experimental work and simplified computational fluid dynamic (CFD) and mathematic modelling [135,137–140] is the following generalized pattern. First, inspired air flows past the ventrobronchi (medioventral secondary bronchi), which are the first airways to branch off of the intrapulmonary primary bronchus, bypassing them (by virtue of the inspiratory valve), and flows either into the dorsobronchi (mediodorsal secondary bronchi, and thence into the parabronchi and the ventrobronchi) or directly into the caudal sacs (caudal thoracic and abdominal). Then, during expiration, instead of flowing out of the intrapulmonary primary bronchus, the air in the caudal sacs is directed into the dorsobronchi and, subsequently, into the paleopulmonic parabronchi and out through the ventrobronchi, with the hypothesized expiratory valve proposed as functioning to prevent the direct, retrograde (tidal) flow back through the primary bronchus ([135]; for reviews on unidirectional airflow, see [66,141]). Unidirectional airflow in the avian lung thus requires the dual presence of the inspiratory aerodynamic valve, situated at the interface of the ostia of the ventrobronchi and intrapulmonary primary bronchi, and the expiratory aerodynamic valve, located at the end of the intrapulmonary primary bronchus [133,135,141]. A smooth muscle constriction just proximal to the ventrobronchi termed the *segmentum accelerans* has been identified in chickens (*G. gallus*), geese (*Anser anser*) and pigeons (*Columba livia*), which may impact the efficacy and function of the inspiratory valve [142,143]. A few experimental studies have shown that avian airflow patterns may deviate from the standard pattern, but it is not clear whether or not this is due to partial failure of the valve, anatomical ambiguities, panting or other unknown physiologic aspects of avian respiration [13,144].

Relative to the inspiratory valve, the expiratory valve is much less well understood [141]. A series of physical models demonstrated that the inclination/angulation of the airways are important relative to the direction/course of air flow [145], that the velocity of airflow may also be important in maintaining the valve [146] and that the valve itself may function differently under different respiratory parameters in live animals and its exact position remains ambiguous [129]. The impact of air sacs on flow patterns also remains ambiguous. Surgical experiments in which the cranial thoracic, caudal thoracic and abdominal air sacs were variably occluded in chickens (*G. gallus*) indicated that interference with these sacs did not disrupt aerodynamic valving (i.e. unidirectional airflow patterns) within the gas-exchanging lung [147]. That is, airflow patterns were not found to be impacted by the distribution, location or number of cranial, caudal or abdominal air sacs [147]. However, these experiments are much more difficult to conduct on the interclavicular and cervical sacs due to their deeper connections with the ventrobronchi [16,147]. Despite decades of work, the mechanisms underpinning the evolution and function of inspiratory and expiratory valves in birds remains elusive. Additionally, interspecific variation in air flow patterns within Aves is largely an unexplored area of research.

Confounding full understanding of unidirectional airflow is the recent discovery of this trait in crocodilians [22,46,67], varanids [148] and iguanas [149]. While crocodilians have secondary bronchi that have been proposed to be homologous to those of birds [7,64], and show similar caudal-to-cranial airflow patterns in these specific airways [22,46], they do not have any extra-pulmonary air sacs like their avian relatives. More specifically, the initial secondary bronchus in crocodylians is proposed to be homologous to avian ventrobronchi, and the subsequent large secondary bronchi homologous to the dorsobronchi, due to both similarities in anatomy and airflow patterns [7,22,46,67]. Like birds and crocodilians, varanids possess multi-bronchial (= multichambered or multicameral) lungs [126,148] (figure 1*g*,*h*), and while unidirectional airflow patterns in these animals are distinctly different from crocodilians and birds, they also possess aerodynamic valves [148,150]. Green iguanas (*Iguana iguana*) have also been found to maintain unidirectional airflow via a valving mechanism, but they have simple lungs (two offset stacked chambers or bronchi with a shared ostium) and use a form of jetting where the air circulates in the same direction during both phases of the respiratory cycle [149].

Given this distribution of pulmonary features, it has been proposed that unidirectional airflow coupled with aerodynamic valves are ancestral for sauropsids and that early taxa may have benefited from this pulmonary trait under conditions of hypoxia [28,66,141]. Farmer has additionally proposed that unidirectional flow may have evolved in concert with a form of cardiogenic flow facilitating gas-exchange during apnea [28,66]. As a result, the long-standing hypothesis that unidirectional airflow evolved to facilitate flight and the elevated metabolic demands of avian endothermy has been overturned. While the most parsimonious hypothesis at present is that unidirectional flow is ancestral for Sauropsida, stark differences in airflow patterns, particularly between archosaurs (i.e. crocodilians and birds) and other sauropsids, and the limited data on flow pattern variation across Sauropsida, render ancestral reconstructions of specific pulmonary soft tissues and airflow patterns impossible.

### (c) Post-cranial pneumaticity and pneumatizing diverticula

Post-cranial skeletal pneumaticity (PSP) is the invasion of pulmonary epithelia into adjacent skeletal tissues. In birds, numerous diverticula extend from the gas-exchanging parenchyma and ventilatory air sacs to variably invade and pneumatize the post-cranial skeleton in most species of birds [69,90–92,151–156]. PSP is also highly variable, both intra- and interspecifically, and pneumatizing epithelia often invade bones asymmetrically [8,154]. Some of this variability may be associated with sex or physiological differences [156–158], but these hypotheses remain to be properly investigated. PSP is also often reduced in birds that occupy aquatic environments, with a complete secondary loss in some diving birds, including penguins, darters and some diving ducks (e.g. [51,69,93,153,159]). In contrast, PSP can be extensive in some taxa, with nearly every skeletal element pneumatized in e.g. pelicans, storks, hornbills, New and Old World vultures, and screamers [93,96]. PSP develops approximately two months post-hatching based on a few seminal studies on the invasion of pneumatizing diverticula into the axial skeleton of galliform and columbiform birds [160–163]; however, the timing and sequence of pneumatization of specific elements differs between these taxa [91], and very little is known about the intra- and interspecific variation in the development of these pneumatizing diverticula. The impact of PSP on bone is very poorly understood, with only a few studies attempting to directly quantify the impact of pneumaticity on the internal bone architecture and biomechanics of bone in birds (e.g. [158,164]; for a review see Moore and Schachner [91]). PSP is one of the most distinct aspects of the avian respiratory system that attracts considerable attention from an ecological and paleontological perspective, but substantial questions remain regarding its relationship with the pulmonary soft tissues and its biomechanical impact on the avian skeleton.

Although PSP is sometimes described as unique to birds among living vertebrates, there is some evidence that skeletal pneumatization, or a similar phenomenon, occurs in certain lineages of fishes. A few species of osteoglossiform fishes—the butterfly fish (*Pantodon buchholzi*) and the African arowana (*Heterotis niloticus*)—have been described as having post-cranial pneumaticity via the swim bladder. These anatomical observations are superficially consistent with the hypothesis that pulmonary membranes of the swim bladder are capable of invading and dissolving adjacent skeletal tissues [165–169]. However, very little is known regarding the swim bladder–bone interface, and the developmental and cellular mechanisms underpinning their interactions. In particular, it is not clear if this phenomenon constitutes a genuine form of invasive post-cranial skeletal pneumaticity that is analogous to that in birds, or if instead diverticula of the swim bladder come to occupy pre-existing vacuities in the vertebrae that form without a process of pneumatization. The latter possibility is supported by the observation that such vertebral vacuities are taxonomically widespread across fishes (e.g. [170]), including many species that apparently lack invasive diverticula of the swim bladder [165,166]. This was even posited by Icardo *et al*. [165] who observed that in *H. niloticus*, the apneumatic caudal vertebrae possess vacuities that are extremely similar to those occupied by the swim bladder in the trunk vertebrae, suggesting that the bladder may have opportunistically invaded pre-existing open spaces already present in the trunk vertebrae. Nevertheless, additional research is needed to characterize putative PSP in *P. buchholzi* and *H. niloticus*. Pending further investigation, it is most conservative at present to state that PSP is unique to birds among *extant tetrapods*.

### (d) The origin of the functionally decoupled avian pulmonary system in extinct non-avian sauropsids

A fully ‘avian-style’ respiratory system has been reconstructed in pterosaurs and non-avian theropod and sauropod dinosaurs to varying degrees (e.g. [93,171–175]). There are two well-established osteological correlates for pulmonary tissues in extinct ornithodirans. The first is the presence of forked dorsal (thoracic) ribs, which necessarily incise and immobilise the gas-exchanging lung. Schachner *et al*. [40,41] first established that such ribs could be used to reconstruct regions of immobilized pulmonary parenchyma in extinct ornithodirans, and this correlate has since been quantitatively validated by Brocklehurst *et al*. [39]. The second osteological correlate of specific pulmonary soft tissues is the presence of PSP, which has been well-described in extinct archosaurs (e.g. [79,92,93,176–178]) and signifies the existence of a highly heterogenous lung with invasive diverticula. O'Connor & Claessens [172] and O’Connor [92] argued that specific post-cranial bones are only invaded by diverticula of particular components of the lung in extant birds. The proposed universality of these patterns has been used as grounds to reconstruct pulmonary anatomy in extinct ornithodirans. Specifically, the presence of unambiguous correlates of PSP in the putative ‘territories’ pneumatized in extant birds by cervical and abdominal air sac diverticula has been used to reconstruct homologs of these air sacs in neotheropods (e.g. *Majungasaurus* [93,172]), neosauropods (e.g. *Haplocanthosaurus* [178]) and ornithocheirid pterosaurs (e.g. *Anhanguera* [171]).

The interpretation and reconstruction of these specific pulmonary tissues based on the presence of PSP can be challenged on two fronts. First, a few species of osteoglossiform fishes have been described as having post-cranial skeletal pneumaticity via the swim bladder ([165–169]; see above). Although additional research is required to establish a process of skeletal pneumatization in these fishes, the possibility that PSP occurs in a pulmonary system so dramatically distinct from that of birds underscores the need for caution in reconstructing specific soft tissues in species known only from defleshed skeletons. Second, recent data from the common ostrich (*S. camelus*) demonstrate the existence of pneumatizing diverticula extending directly from the gas-exchanging lung and *only* from the lung to pneumatize all pneumatic bones of the post-cranial skeleton [7]. This observation calls into question the current paradigm, which describes sac-specific patterns of skeletal pneumatization as universally fixed across all birds and, by extension, their extinct ornithodiran relatives.

These data show a clear need for systematic investigation into the individual pulmonary tissues that are providing the source of pneumatization across Aves. If other species of birds prove upon re-examination to deviate in diverse ways from the rules proposed by O’Connor & Claessens [172] and O’Connor [92], then it may not be possible to reconstruct specific air sacs and diverticula in extinct taxa. High-fidelity documentation of pneumatizing diverticula in extant birds via µCT scan segmentation, latex injection and dissection is an active area of research by the authors. More broadly, there is a pressing need for greater understanding behind the molecular and developmental mechanisms influencing post-cranial skeletal pneumatization in vertebrates. It is possible that post-cranial pneumatization in ornithodiran lineages is distinct from whatever phenomenon occurs in *Pantodon* and *Heterotis*, but this remains to be demonstrated by comparative developmental and molecular studies. Moreover, such work may provide novel insights on the nature of ambiguous correlates of pneumaticity (e.g. lamina, fossae) in non-ornithodiran archosaurs and other sauropsid taxa, and on why pneumaticity does not occur with the mammalian bronchoalveolar lung but does occur via the upper respiratory tract, where paranasal and paratympanic diverticula invade certain cranial elements (i.e. craniofacial sinuses—e.g. Witmer [179,180]).

In addition to the two well established correlates of pulmonary anatomy, there is a third osteological correlate that has largely been left out of the discussion with respect to reconstructing lungs in extinct archosaurs: the horizontal septum (figure 2). As discussed above, the horizontal septum is vitally essential to the normal functioning of the avian lung, and its evolution was a critical step in producing a fully functionally decoupled respiratory system [31,61]. Without evidence of a horizontal septum, it is not possible to reconstruct an ‘avian-like’, fully functionally decoupled lung in extinct saurischian dinosaurs or pterosaurs. However, it should be possible to find osteohistological evidence for this septum, as the costopulmonales muscles (which act on the horizontal septum) attach to the medial aspects of the dorsal ribs in birds [20] and to our knowledge are absent in non-avian sauropsids, although this has yet to be tested in extinct taxa. Without evidence for the horizontal septum and costopulmonales muscles, the lungs of extinct ornithodirans with forked ribs and PSP fall short of being fully ‘avian-like’. Instead, they may have been dorsally immobilized, with a large flexible, ventral saccular region. Pneumatizing diverticula may have emerged either directly from the exchange parenchyma or from the caudoventral region, with elaborate diverticula that wrapped around the abdominal viscera, as occurs not only in birds but also in varanids and chameleons (figure 1*f*–*h*).

## 5. What is next for avian lungs?

What makes a bird lung a bird lung? In some ways, they can be viewed as highly specialized lungs, modified from the original reptile *bauplan*. Their extinct relatives have been demonstrated to have evidence of post-cranial pneumaticity and pulmonary heterogeneity, and both pulmonary diverticula and unidirectional airflow are common traits across much of Sauropsida. Along with the cross-current gas-exchange system, it is the full functional decoupling of the ventilator from the gas-exchanger (subdivided by the horizontal septum) that is one of the more robust anatomical traits that differentiates avian lungs from other sauropsids and that facilitated the evolution of a lung capable of e.g. efficient gas exchange at high altitudes [181].

What is missing of our knowledge of avian lungs is a full understanding of the diversity and variation in both structure and function of the pulmonary tissues across Aves. In other words, true comparative and quantitative ecomorphological analyses have yet to occur, although some ecological and physiological studies provide tantalizing glimpses of evolutionary differentiation of the avian pulmonary system. For example, experimental work on diving tufted ducks (*Aythya fuligula*) and little penguins (*Eudyptula minor*) as well as non-diving black-billed magpies (*Pica pica*) has shown that locomotory behaviour can function to enhance the movement of gases through the respiratory system via the compression of the air sacs (e.g. [182–185]). What roles air sacs play both in ventilatory and non-ventilatory behaviours in birds still needs to be teased apart. Additionally, the expiratory valve is an open area that has yet to be fully explored with modern experimental approaches like CFD modelling. Hypotheses regarding pulmonary tissue homologies across all of Sauropsida have not been tested, and the evaluation of robust testable osteological correlates remains an open question.

## Data Availability

The imaging data associated with the Cuvier’s dwarf caiman are freely available via Data Dryad: [186]. The imaging data associated with the ostrich are available via Data Dryad here: [187]. The imaging data associated with the African grey parrot used in this are available here: https://www.morphosource.org/concern/media/000553437?locale=en. The red-tailed hawk and great horned owl imaging data are currently being used for ongoing lab and student projects and are available upon request.
